# Procedural sedation competencies: a review and multidisciplinary international consensus statement on knowledge, skills, training, and credentialing

**DOI:** 10.1016/j.bja.2024.07.036

**Published:** 2024-09-25

**Authors:** Piet L. Leroy, Baruch S. Krauss, Luciane R. Costa, Egidio Barbi, Michael G. Irwin, Douglas W. Carlson, Anthony Absalom, Gary Andolfatto, Mark G. Roback, Franz E. Babl, Keira P. Mason, James Roelofse, Paulo S. Costa, Steven M. Green, International Committee for the Advancement of Procedural Sedation

**Affiliations:** 1 Department of Pediatrics, Maastricht University Medical Centre and School of Health Professions Education, Maastricht University, Maastricht, The Netherlands; 2 Department of Pediatrics, Harvard Medical School, Boston Children's Hospital, Boston, MA, USA; 3 Department of Pediatric Dentistry, Federal University of Goias, Goiania, Goias, Brazil; 4 Department of Pediatrics, Institute for Maternal and Child Health-IRCCS ‘Burlo Garofolo’, Trieste, Italy; 5 Department of Anaesthesiology, University of Hong Kong, Queen Mary Hospital, Hong Kong; 6 Department of Pediatrics, Southern Illinois University School of Medicine, Springfield, IL, USA; 7 Department of Anaesthesia, University of Groningen, University Medical Center Groningen, Groningen, The Netherlands; 8 University of British Columbia Department of Emergency Medicine, Lions Gate Hospital, North Vancouver, British Columbia, Canada; 9 Department of Pediatrics, University of Colorado School of Medicine, Aurora, CO, USA; 10 Departments of Paediatrics and Critical Care, University of Melbourne, Emergency Department, Royal Children's Hospital, Emergency Research, Murdoch Children's Research Institute, Parkville, WA, Australia; 11 Department of Anesthesia, Harvard Medical School, Boston Children's Hospital, Boston, MA, USA; 12 Departments of Anaesthesia, University of the Western Cape, Stellenbosch University, Tygerberg, Republic of South Africa; 13 Department of Pediatrics, Federal University of Goias, Goiania, Goias, Brazil; 14 Department of Emergency Medicine, Loma Linda University, Loma Linda, CA, USA

**Keywords:** competencies, credentialing, entrustable professional activity, medical education, privileging, procedural sedation, quality and patient safety

## Abstract

Procedural sedation is practised by a heterogeneous group of practitioners working in a wide array of settings. However, there are currently no accepted standards for the competencies a sedation practitioner should have, the content of sedation training programmes, and guidelines for credentialing. The multidisciplinary International Committee for the Advancement of Procedural Sedation sought to develop a consensus statement on the following: which competencies should medical or dental practitioners have for procedural sedation and how are they obtained, assessed, maintained, and privileged. Using the framework of Competency-Based Medical Education, the practice of procedural sedation was defined as a complex professional task requiring demonstrable integration of different competencies. For each question, the results of a literature review were synthetised into preliminary statements. Following an iterative Delphi review method, final consensus was reached. Using multispeciality consensus, we defined procedural sedation competence by identifying a set of core competencies in the domains of knowledge, skills, and attitudes across physical safety, effectiveness, psychological safety, and deliberate practice. In addition, we present a standardised framework for competency-based training and credentialing of procedural sedation practitioners.

**Editor's key points**

• There are currently no accepted standards for the competencies required for a sedation practitioner, the content of sedation training programmes, and guidelines for credentialing**.**
• A multidisciplinary team of experts convened to develop a consensus statement regarding the competencies that practitioners should have for procedural sedation and how are they obtained, assessed, maintained, and privileged.
• The results of a literature review were synthetised into preliminary statements that were refined though an iterative Delphi review method, which led to identification of a set of core competencies in the domains of knowledge, skills, and attitudes across physical safety, effectiveness, psychological safety, and deliberate practice.
• A standardised framework for competency-based training and credentialing of procedural sedation practitioners was developed and is presented here.
• This consensus statement forms the basis for standardisation of training and to inform guidelines and regulations regarding training, privileging, and credentialing of procedural sedation practitioners to further enhance the safety and quality of care for patients undergoing procedural sedation.

Defining and assessing competence is a fundamental issue within the multidisciplinary field of procedural sedation. Although procedural sedation is performed by a diverse group of practitioners in a wide array of settings, it is characterised by a common set of principles, objectives, endpoints, and procedures. It is a complex task with serious potential risks that requires the integration of an extensive set of knowledge, skills, and attitudes.

Specific questions regarding competence have, thus far, defied well-accepted answers: Who is qualified to provide procedural sedation? Which competencies (i.e. knowledge, skills, and attitudes) are required in order to be competent? What constitutes adequate training? How are competencies assessed, approved, and maintained? Most specialties whose members regularly perform procedural sedation have their own clinical practice guidelines.^1^, ^2^, ^3^, ^4^, ^5^, ^6^, ^7^ However, these guidelines often fail to specify the required competencies and rarely address education, training, or credentialing requirements for practitioners.^1^^,^^8^ In addition, specialty-specific training programmes have been inconsistent in educational approaches, supervision, practical exposure, and evaluation practices.^9^ A consensus statement is therefore required.

The International Committee for the Advancement of Procedural Sedation (ICAPS, www.proceduralsedation.org) is an independent, international, multidisciplinary forum to facilitate consensus generation between experts in the area of procedural sedation. Using a Competency-Based Medical Education (CBME) framework, our objectives were to define the minimum competencies (i.e. knowledge, skills, attitudes) for all medical and dental procedural sedation practitioners upon which a standardised framework for competency-based curricula can be established. By considering procedural sedation as a complex professional task and exploring it from the perspective of its constituent competencies, we focus on the key question of to whom procedural sedation can be responsibly entrusted.

## Methods

### Scope

Consistent with the principles of CBME, we formulated standard definitions of competencies in procedural sedation practice for all practitioners, regardless of specialty training, setting, or patient age. We excluded patient selection and screening, monitoring, drugs, and other practice issues, which are well covered in existing clinical practice guidelines.^1^^,^^2^^,^^7^^,^^10^ We also excluded facility accreditation from the scope of this document as it is subject to governmental and regulatory oversight.^11^

### Definitions

Definitions for key terms are shown in Table 1.

**Table 1:** Terminology.

**Fig 1:**
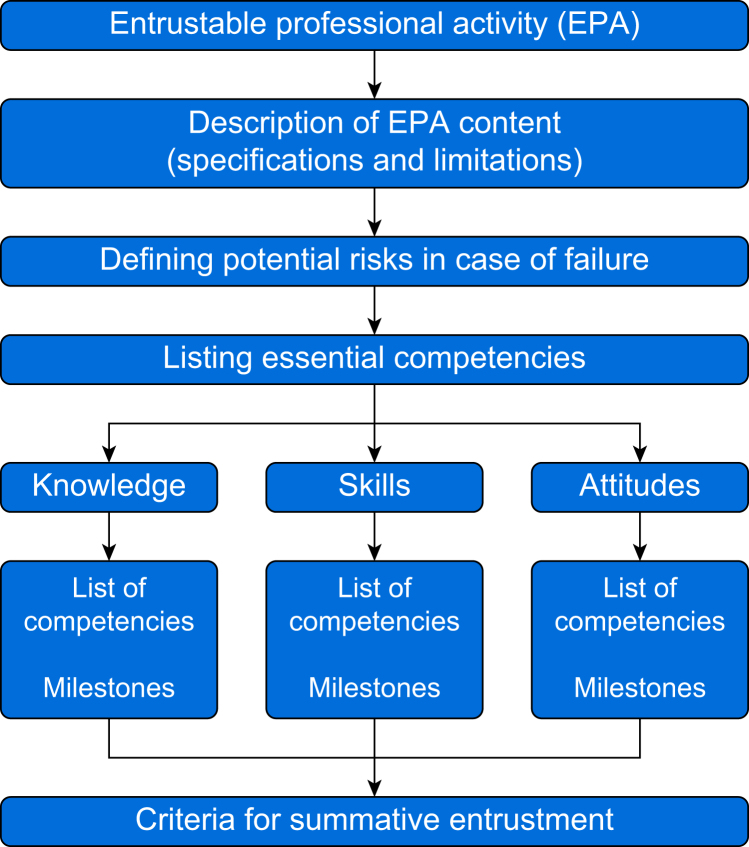
Framework for describing an Entrustable Professional Activity.^27^.

*Competence:* An individual's ability to perform a professional task or role in accordance to established professionals standards. Competence implies achievement of a minimum set of knowledge, skills, values, and attitudes that together contribute to an acceptable level of performance.
*Competency*: An observable ability of a health professional such as knowledge, motor and cognitive skills, values, or attitudes. As competencies are observable and measurable, they can be used to support and verify the progress of the learner towards competence.^12^, ^13^, ^14^
*Competency assessment*: In Competency-Based Medical Education (CBME), assessment serves both as an objective judgement of performance (assessment of learning) and a ‘teachable moment for further improvement’ (assessment for learning). Self-assessment combined with direct, repeated, and constructive feedback by experts coupled with formative assessment (ongoing feedback during the course of an assessment) has been shown to foster competence development and professional growth.^15^
*Competency-Based Medical Education (CBME):* An approach to the design, implementation, assessment, and evaluation of a medical curriculum, based on a set of competency outcomes—the abilities to function as an effective health professional.^12^^,^^13^^,^^16^ In CBME, competencies are explicitly sequenced to support the learner's progression. The time needed to attain the intended outcomes varies among learners and settings.^13^^,^^17^ Learning experiences are tailored to these outcomes and should resemble the authentic practice environment. Instruction formats are competency-based, meaning that teaching is focused on learning through experience and application and not just knowledge acquisition. Teachers function by instructing practitioners using actionable feedback. In CBME, competency assessment is based on multiple sources, including workplace observation with meaningful and individualised feedback. Progression towards competence is based on formal entrustment decisions and not on the time spent on learning.^16^
*Credentialing*: The methodology used to validate a professional's credentials to participate in patient care through assessing registration, certification, licensure, admission to association membership, the award of a diploma or degree, and evidence of ongoing medical education (MESH terminology – National Library of Medicine).^18^
*Deliberate practice*: An educational concept that supports a practitioner to acquire over time and maintain mastery within a given domain.^19^^,^^20^ Deliberate practice refers to repetitive domain-specific performance that challenges the learner and provides opportunities for informative, proximate feedback.^21^, ^22^, ^23^, ^24^ Deliberate practice can be organised through a structured program of continuing professional development, including simulation training, observed clinical practice, or both.
*Entrustable Professional Activity* (EPA): A unit of professional practice that can be fully entrusted to a learner to execute ultimately in an unsupervised manner.^25^ EPAs are discrete tasks (e.g. managing deep sedation in a patient with ASA physical status 2), or bundles of tasks (e.g. managing a procedural sedation service) that are independently executable, observable, and measurable in their process and outcome, and, therefore, suitable for documenting progress, making entrustment decisions and credentialing.^12^^,^^26^ EPAs are embedded in a clinical context and operationalise CBME through a stepwise and safe engagement of trainees in clinical practice, linking progressive competence to progressive autonomy in patient care.^27^
*Milestone:* A behavioural descriptor that marks a level of performance for a given competency.^28^
*Privileging*: The formal act of authorising and entrusting specific healthcare practitioners to perform procedural sedation unsupervised by their responsible authority, such as director, hospital, board, college, and government entity.^18^
*Portfolio*: A tool for collecting and managing multiple forms of assessment that demonstrate how learners are fulfilling tasks and progressing towards developing competence. Portfolios report on work done, feedback received, progress made, and plans for improving competencies, and provide a source of input for final entrustment decisions and credentialing.^29^
*Procedural sedation and analgesia (procedural sedation)*: The administration of one or more pharmacological agents to facilitate a diagnostic or therapeutic procedure while targeting a state during which airway patency, spontaneous respiration, protective airway reflexes, and haemodynamic stability are preserved while alleviating anxiety and pain.^30^
*Summative entrustment*: The formal and deliberate determinations of the amount of supervision a learner needs. This decision results in defining what learners are formally allowed to do at a given level of supervision. The framework for describing an EPA results in clear criteria for summative entrustment (Fig. 1).

### Educational framework

We consider the practice of procedural sedation as a set of Entrustable Professional Activities (EPAs). EPAs represent the clinical activities that competent practitioners will ultimately practise independently, and help clarify which individual behaviours (i.e. competencies) learners should master to execute these activities successfully. One of the characteristic features of an EPA is that its performance requires integration of competencies, usually across domains.^26^ Structuring a CBME curriculum using a set of EPAs avoids the risk that the mere possession of a set of necessary competencies in isolation would be sufficient to deserve the status of ‘being competent for a given task’. For example, the separate acquisition of knowledge and skills in airway management, advanced life support, and procedural sedation pharmacology is not, in our view, sufficient to be competent to practise procedural sedation. Competence should, therefore, be based on the ability to perform the whole activity of procedural sedation to a predefined level. The final level of achievement for this outcome (i.e. unsupervised practice of all EPAs) is the same for all, although the time to achieve it might vary. EPAs are operationalised by defining their precise content, potential risks in case of failure, relevant core competency domains, constituent knowledge, skills, and attitudes, assessment and criteria for entrustment decisions (Fig. 1).^27^^,^^31^

### Sedation categories

We formulate competencies for all forms of procedural sedation and differentiate between two distinct sedation categories: (1) dissociative, or moderate to deep sedation, and (2) minimal sedation. Strategies for dissociative, or moderate to deep sedation, seek to depress the patient's level of consciousness. As a consequence, they share the same core competencies needed to effectively achieve and maintain a desired sedation level, and recognise and manage potential adverse events. During minimal sedation, the pharmacological agent is an anxiolytic adjunct to a set of non-pharmacologic and analgesic interventions (i.e. topical and local analgesia). Here, the primary goal is not decreasing the level of consciousness but rather achieving optimal patient comfort while enhancing cooperation, collaboration, and trust.^32^, ^33^, ^34^, ^35^, ^36^, ^37^

### Committee selection

Our panel comprised existing members of ICAPS, all previously selected for their established expertise as researchers and leaders in procedural sedation. The mission of ICAPS is to provide an independent, international, multidisciplinary forum to facilitate open dialogue and consensus generation, and to promote optimal, evidence-based, safe, and effective practices for global procedural sedation in patients of all ages. ICAPS is an independent, self-funded entity, with no formal relationship with or sponsorship from industry, specialty societies, constituencies, or other organisations. It includes members from nine countries on six continents, with representation from anaesthesia, critical care, dentistry, emergency medicine, gastroenterology, hospital medicine, and paediatrics. Potential conflicts of interest for members are declared at http://proceduralsedation.org/conflicts-of-interest/. ICAPS has prior experience in writing guidelines and statements.^30^^,^^38^, ^39^, ^40^, ^41^ Fourteen of the 18 ICAPS members agreed to participate in this specific project.

### Literature searches

We performed targeted searches of the PubMed database using combinations of the following keywords/phrases/MESH terms: sedation, conscious sedation, moderate sedation, dissociative sedation, deep sedation, competency-based education, clinical competence, professional competence, professional skills, clinical skills, and credentialing. We limited all searches to human studies from sources in languages spoken and written by our members (Chinese, Dutch, English, French, German, Italian, Portuguese, and Spanish) published between January 2000 and September 2023.

### Project organisation

In developing this statement, we adhered to the principles and methodology advocated by the US National Academy of Medicine (formerly the Institute of Medicine)^42^ and other prominent sources,^43^, ^44^, ^45^ and as quality checked by the National Guideline Clearinghouse Extent of Adherence to Trustworthy Standards instrument.^46^

### Evidentiary quality

There is extensive literature documenting a high safety profile for procedural sedation when performed by a variety of practitioners.^47^ However, there is little or no compelling evidence on how competence was specifically established for various types of procedural sedation practitioners. A systematic review of this topic noted that ‘No prospective controlled studies were found comparing different levels of professional competence and the effectiveness of procedural sedation’.^48^ We recognised that the bulk of this project would rely upon the consensus opinion of our members, and that this would be the principal limitation of the effort.

### Delphi review

The project period extended from July 2021 to February 2024. We began with a general, open-ended survey of committee members regarding optimal reporting format and content using the nominal group technique. This feedback was recirculated among the panel in anonymised manner with repeat feedback until theme saturation was reached.

A committee task force composed, and then circulated, a preliminary outline and working drafts of statement segments. We then initiated a sequential consensus generation process using the Delphi method with an iterative series of e-mail surveys and draft critiques. After each round, the responses from members were displayed to all in an anonymous manner. The committee members could then revise their earlier responses based upon ongoing feedback, with our co-chairs serving as moderators to guide the direction of consensus discussion. Ultimately, 12 Delphi cycles were required to achieve consensus.

Upon evidence of nearing consensus, the committee members were asked to respond to the question ‘Does this updated draft segment represent the best possible ICAPS statement on this aspect of sedation competence?’ using a five-point Likert scale: strongly disagree, disagree, no strong opinion, agree, and strongly agree. We quantified our consensus using the following thresholds of either ‘agree’ or ‘strongly agree’: strong (>90%), satisfactory (>80%–90%), moderate (>70%–80%), weak (>60%–70%), or absent (60% or less).

## Results

### What are the minimum competencies required for practicing procedural sedation?

*We reached strong consensus (13/14 strongly agree, 1/14 agree) about minimum competencies for procedural sedation.*

We developed a comprehensive list of minimum competencies based on an ICAPS policy statement on procedural sedation skills,^41^ related multidisciplinary efforts,^2^^,^^7^^,^^10^^,^^47^, ^48^, ^49^, ^50^, ^51^, ^52^, ^53^ and studies on the impact of adherence to established guidelines and the implementation of competency-based curricula.^54^, ^55^, ^56^, ^57^, ^58^ According to established outcomes for procedural sedation-related quality,^50^^,^^59^^,^^60^ we stratified these competencies into three domains (Table 2): patient safety (i.e. avoiding patient harm and minimising sedation-related risks), effectiveness (i.e. assuring optimal procedural success and patient comfort), and psychological safety (i.e. assuring the patient's emotional and psychological well-being).

**Table 2:**
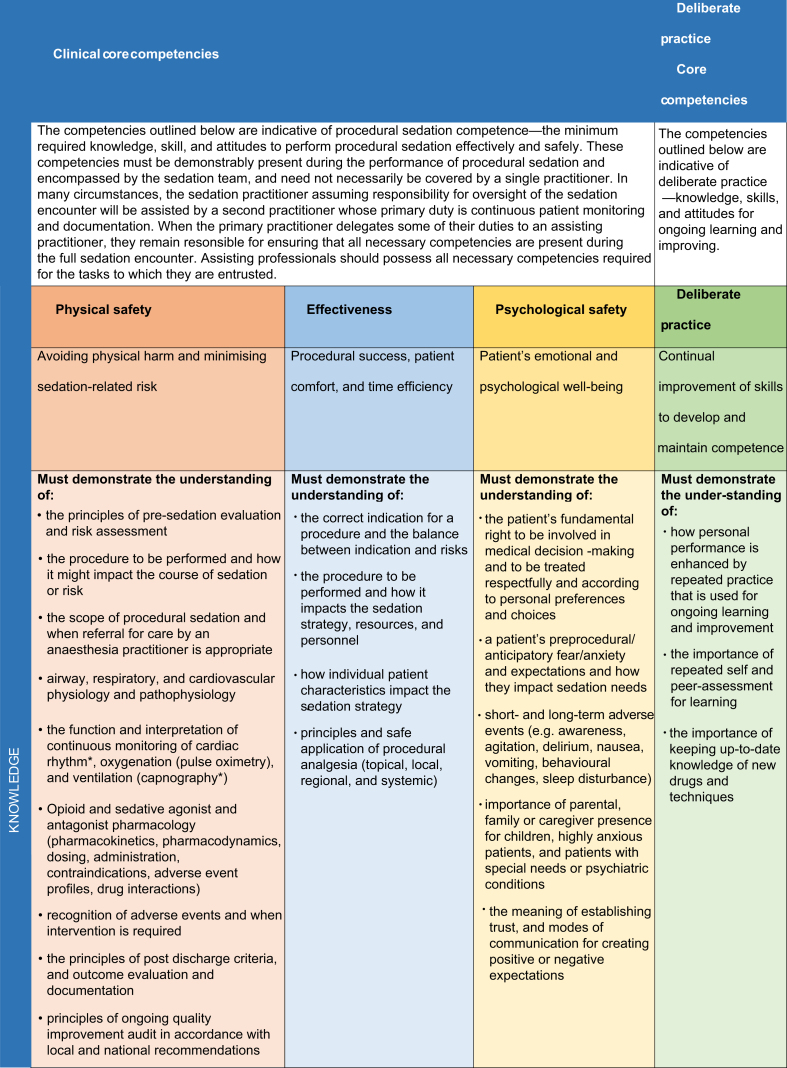

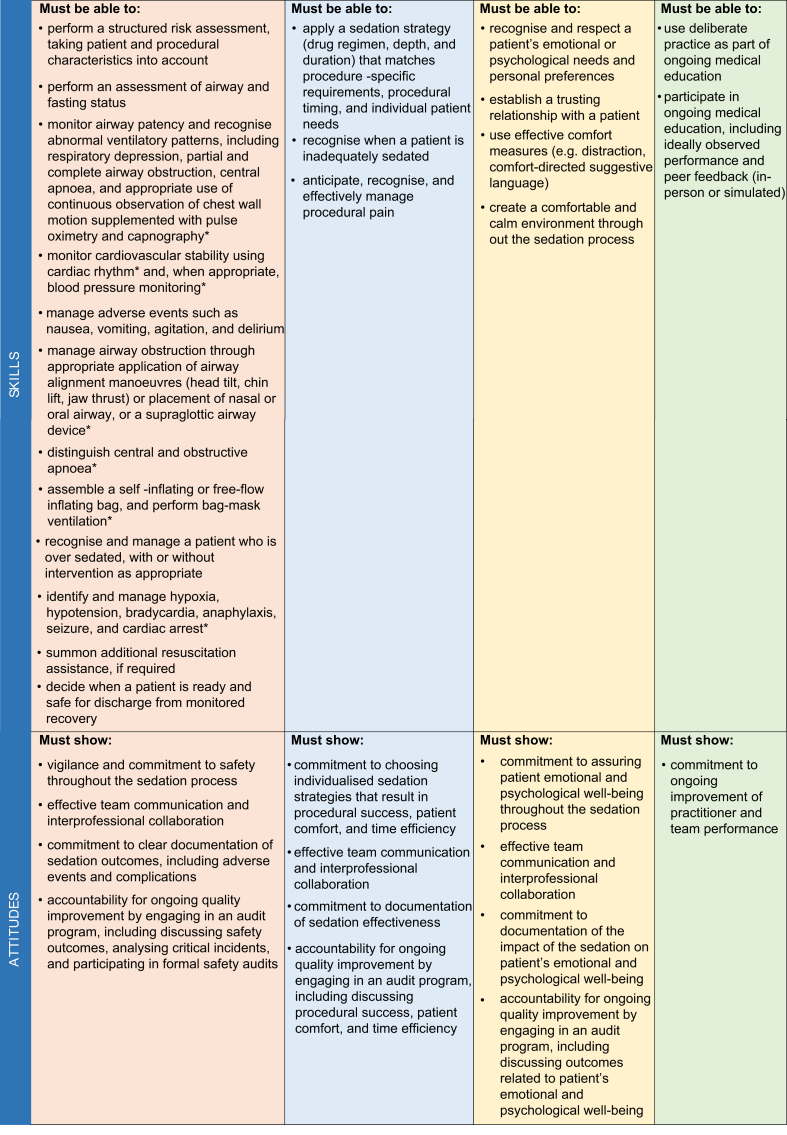
Procedural sedation competencies. ∗Less relevant competencies for practitioners performing minimal sedation in low-risk patients.

### How is procedural sedation competence obtained?

*We reached strong consensus (13/14 strongly agree, 1/14 agree) about how procedural sedation competence should be obtained.*

Practitioners, after completing a general medical or dental degree, can become competent in procedural sedation as part of a specialty training programme, or through a certified procedural sedation training programme. In both cases, performing procedural sedation is considered a complex professional task requiring integration of multiple competencies (Table 2). Therefore, CBME is an optimal framework for the design of a procedural sedation curriculum.^16^ Structuring this curriculum according to a set of EPAs helps trainees and educators gain insight into progressive acquisition of competencies and make entrustment decisions.^61^^,^^62^ Different categories of EPAs can be distinguished:

*Clinical EPAs related to the practice of procedural sedation*: The intended learning outcomes of a particular procedural sedation curriculum (e.g. minimal sedation in a child, propofol-based sedation in adults for endoscopy, ketamine-based sedation for painful procedures in patients with ASA physical status 1 or 2) determine the content and relevance of the contributing clinical EPAs. Published CBME curricula can inform curriculum designers to identify and describe the relevant clinical EPAs for their procedural sedation training programme (Supplementary material 1).^31^^,^^63^^,^^64^

*Nested EPAs*: Within a clinical EPA, several nested EPAs might be identified, that is, self-contained smaller units of practice that have their own specific set of competencies and can be trained separately. Examples are airway management, basic and advanced life support, pharmacology, use of local and topical analgesia, patient communication, and establishing trust.^12^

*Organizational EPAs* include oversight of the sedation process as it relates to clinical need, staffing and availability of resources, and maintenance of standards through adherence to established guidelines,^55^^,^^56^ quality management (i.e. outcome reporting and reviewing, including adverse events and efficiency),^38^^,^^65^ and continuing medical education.^66^ Although these EPAs might be more relevant for directors of sedation programmes, they are part of the overall procedural sedation competence framework as they contribute substantially to procedural sedation quality.

A procedural sedation curriculum should encompass a variety of educational modalities: knowledge acquisition through self-study or didactic instruction (e.g. lecture, video, demonstration, third-party training modules), application of knowledge, and demonstration of clinical skills (e.g. through simulation modelled as an Objective Structured Clinical Examination and supervised sedation), with exposure to different patient morbidities and the full range of procedures that the future practitioner is expected to manage.^67^^,^^68^ Each of these formats should involve structured feedback and include a portfolio to document and monitor the learner's progression towards proficiency (Table 1). Final privileging is based on documented exposure to, and demonstrated competence in, a specific category of sedation techniques, procedure, and patient types, as defined within the curriculum's intended learning outcomes.

### How is procedural sedation competence assessed?

*We reached strong consensus (14/14 strongly agree) about how procedural sedation competence should be assessed.*

Competence in procedural sedation should be assessed through multiple sources, including knowledge evaluation and practical skills demonstrated through supervised sedation practice (Table 3).^69^

**Table 3:** Approaches to assessment of procedural sedation competencies and performance.^15^.

Assessment format	Examples
Knowledge	- Written knowledge tests on procedural sedation-related topics (e.g. pharmacology, sedation risks, procedural distress, procedural sedation history, content of sedation guidelines)
Knowledge application	- Written assignment or essays on specific procedural sedation topics/cases (e.g. risk assessment based on a theoretical case scenario) - Oral questioning on possible patient management - Case evaluation/reflection (written or oral)
Simulated practice	- Simulated procedural sedation scenario (e.g. high-fidelity scenario training) - Objective Structured Clinical Examination (OSCE) - Oral case presentation/discussion - Skills station (e.g. airway management) - Virtual reality or computer-based cases
Clinical practice	- Direct observation in clinical settings - Video observation of real practice - Multi-source feedback (360-degree feedback) - End of rotation evaluation - Patient outcomes data - Personal project review (e.g. quality audit of the practitioner's procedural sedation service; quality improvement project; evidence-based guideline on a specific topic)

EPA-specific competencies and their corresponding assessment can be structured according to established core competencies, such as the Accreditation Council for Graduate Medical Education (ACGME) competency framework.^69^ For each core competency (i.e. medical knowledge, patient care, interpersonal and communicative skills, professionalism, practice-based learning and improvement, and systems-based practice), the corresponding procedural sedation-required knowledge, skills, and attitudes can be mapped.^69^^,^^70^ To document a learner's progress and prepare for entrustment decisions, specific milestones can be defined, describing the level of performance that is expected at each stage of the developmental trajectory to competence. Evaluators should be trained on how to effectively use assessment to objectively judge performance, document personal progress towards proficiency, and stimulate individual learning (Table 1).^20^^,^^71^, ^72^, ^73^, ^74^, ^75^

Assessing procedural sedation competence is an essential part of a procedural sedation auditing and quality improvement programme. Each procedural sedation setting should participate in a rigorous quality improvement programme that audits sedation practice, tracks adverse events, ensures satisfactory documentation and compliance with protocols, and identifies opportunities for improvement. Based upon local healthcare authority structures, this programme could be overseen by the involved clinical department, an institution-wide multidisciplinary sedation committee, or a designated monitor.

Given widespread tracking variation related to a lack of consistent definitions of adverse event terminology, ICAPS developed a standardised tool for Tracking and Reporting Outcomes Of Procedural Sedation (TROOPS, Supplementary material 2). TROOPS documents sedation adverse events, interventions, and outcomes for patients of all ages, and is applicable for all types of sedation practitioners worldwide.^38^ Similar tools have been published.^76^^,^^77^ Quality improvement programmes should, at a minimum, track and review the following sentinel events: unplanned tracheal intubation, need for neuromuscular block, pulmonary aspiration, vasoactive drug administration, need for chest compressions, neurological deficit, or death. The tracking and reviewing of intermediate outcomes is also highly recommended, including need for positive pressure ventilation, reversal agents, oral airway, i.v. fluid bolus or anticonvulsants, insufficient sedation, escalation of care, hospitalisation, practitioner dissatisfaction, and patient/family dissatisfaction.^38^^,^^76^^,^^77^

### How should procedural sedation be privileged?

*We reached strong consensus (14/14 strongly agree) about how procedural sedation should be privileged.*

Privileging refers to the formal decision by an institution's sedation committee or a designated monitor, based on documented competence at the necessary performance levels for all EPAs, that the trainee can be entrusted to perform procedural sedation independently.^78^ Although the exact conditions for privileging can vary between institutions, the following are essential: (1) completion of a formal training programme that covers all essential competencies listed in Table 2 ^79^(2) documentation (e.g. based on the collected outcomes of a multisource assessment); that the necessary performance levels are met for all relevant EPAs.^78^ Some institutions require specific documentation of the type and variety of sedation procedures performed both supervised and independently^80^; and (3) privileges for procedural sedation should be periodically reviewed and renewed by the sedation committee or designated monitor in accordance with requirements set by the institution and regulatory bodies. Decisions related to renewal of privileges should be based on the level and quality of clinical exposure and engagement in continuous professional development.

### How should procedural sedation competence be maintained?

*We reached strong consensus (14/14 strongly agree) about how procedural sedation competence should be maintained.*

Procedural sedation competence is dynamic and contextual and can advance or recede over time, emphasising the importance of effective deliberate practice (Table 3).^19^^,^^20^^,^^37^^,^^71^, ^72^, ^73^, ^74^, ^75^^,^^81^, ^82^, ^83^, ^84^ Therefore, procedural sedation practitioners should be competent in deliberate practice and actively engage in lifelong learning activities intended for continuing professional development (Table 2).^66^ This can include the following activities: (1) participation in relevant conferences, webinars, workshops, seminars, or web-based formats for self-directed learning helps the practitioner stay updated with the latest guidelines, advances, and best practices in procedural sedation^66^; (2) periodic evaluation of performance through observed practice in clinical or simulated settings, accompanied by informative, proximate feedback. Simulation is an essential element and should be used for the training, evaluation, and maintenance of competencies required for managing critical but rare adverse events. Simulation also allows for effective team training of crew resource management, interprofessional communication, and collaboration^8^^,^^85^, ^86^, ^87^, ^88^, ^89^; (3) interdisciplinary meetings and case-based team discussions of critical incidents facilitate identification of areas for improvement and adoption of best practices, enhance teamwork, and promote the exchange of knowledge and expertise^90^; (4) access to updated procedural sedation evidence-based guidelines, protocols, and resources^55^; and (5) participation in procedural sedation-related research can help practitioners to stay informed about new evidence and to gather new information that could inform and potentially change clinical practice.^91^

## Discussion

We present a competency-based educational framework for procedural sedation training and credentialing, grounded in the procedural sedation literature, educational theory, and multispeciality consensus. Based on accepted patient outcomes (i.e. optimal patient safety, effectiveness, psychological and emotional well-being), we identify the knowledge, skills, and attitudes that define procedural sedation competence and the ongoing learning activities for improving and maintaining competence that forms the minimum standard a professional should meet to be entrusted to administer procedural sedation.

We believe that taking this educational perspective is an important strength of our consensus statement. By considering sedation as an EPA, the decision regarding to whom sedation can be safely entrusted must be based on the demonstrable integration of competencies after a formal training programme. Previous sedation guidelines, which are primarily speciality-specific, define the safe practice of sedation (i.e. patient evaluation, selection and preparation, vital sign monitoring, drug selection and pharmacology, recovery care), but do not specify the required competencies nor address education, training, or certification requirements for practitioners.^1^, ^2^, ^3^, ^4^, ^5^, ^6^, ^7^ Our guideline is the first to directly address the issues of training and credentialing in procedural sedation and provides a standardised framework for competency-based curriculum design.

We believe that our work is relevant for patients of all ages. Although the majority of authors have a paediatric background, most of the authors have been involved in adult sedation programmes and adult sedation guideline development and research. In addition, our recommendations are consistent with adult and paediatric studies and guidelines.

Our work has several limitations. Given the professional backgrounds of the authors involved, our consensus generating process was informed by medical and dental expertise. Therefore, our recommendations might be not fully applicable for nurse sedation providers. Nurse-led sedation is subject to local regulations regarding entrustment, independent practice, and degree of supervision. However, evidence suggests that specially trained nurses, working within a well-organised and supervised safety network, are able to administer safe and effective sedation for adults and children. The necessary competencies are essentially the same as presented in this consensus statement.^51^, ^52^, ^53^^,^^92^, ^93^, ^94^, ^95^

A second limitation is that most of the literature relates to North American and European settings, and most panel members provide sedation in high-resource settings. In resource-limited settings, some educational and monitoring resources (e.g. capnography, ECG monitoring, simulation training, quality improvement programmes) might not be available. However, research on sedation safety in resource-limited settings suggests that most of the competencies will be important to provide responsible patient care.^92^^,^^96^, ^97^, ^98^ Further, concepts such as psychological safety and emotional well-being vary in content between different cultural conditions.

Finally, a Delphi method has specific limitations (e.g. participation selection bias, overreliance on expertise) that might impact the validity and reliability of final consensus statements. Nevertheless, we believe that the process was fair, transparent, and demonstrated a measurable degree of final consensus.

This consensus statement is intended to form the basis for standardisation of training and to inform guidelines and regulations regarding training, privileging, and credentialing of procedural sedation practitioners. As such, it contributes to further enhancement of the safety and quality of care in patients undergoing procedural sedation.

## Authors’ contributions

Formulation of study concept and design, literature review, coordination of Delphi analyses, drafting of the manuscript, and critical review of the manuscript: PLL, BSK, SMG

Methodologist for the project: SMG

Critical feedback at each Delphi cycle: LRC, EB, MGI, DWC, AA, GA, MGR, FEB, KPM, JR, PSC

Approval of the final version of the manuscript: all authors

## Declarations of interest

AA has performed sponsored phase 1 research for The Medicines Company and Rigel Pharmaceuticals. He has performed paid consultancy work for Johnson and Johnson, The Medicines Company, Becton Dickinson, Ever Pharma, Philips, and PAION (paid to institution). His research group has received unrestricted research grants from Orion, Becton Dickson, and Drager (paid to institution).

FEB has received research grants from the National Health and Medical Research Council, Canberra, Australia, and the Royal Children's Hospital Foundation, Melbourne, Australia.

LRC has received research grants from the following Brazilian foundations: Conselho Nacional de Desenvolvimento Científico e Tecnológico (CNPq), Coordenação de Aperfeiçoamento de Pessoal de Nível Superior (CAPES) e Fundação de Amparo à Pesquisa do Estado de Goiás (FAPEG).

PSSC was supported by the Brazilian National Research Scientific Research Council.

MGI has received travel support from Fresenius Kabi for attending an advisory board meeting and giving a lecture on intravenous anaesthesia (propofol). His department receives academic support for a questionnaire survey on barriers to TIVA use.

KPM has received support from Hospira for investigator initiated studies and unrestricted educational support for conferences.

The other authors have no conflicts to declare.
