# Supplementary material for: Procedural sedation competencies: a review and multidisciplinary international consensus statement on knowledge, skills, training, and credentialing
Source: Br J Anaesth. 2024 Sep 25;134(3):817–29. doi: 10.1016/j.bja.2024.07.036 (PMC11867087; doi:10.1016/j.bja.2024.07.036)
Supplement: Multimedia component 1 [file mmc1.docx]

**Supplementary material 1: Examples of a set of clinical Entrustable Professional Activities related to a specific type of procedural sedation practice**

To illustrate possible EPAs and their content description for a given training (sub)program. Entrustable Professional Activities should be described by defining their precise content, potential risks in case of failure, relevant core competency domains, constituent knowledge, skills and attitudes, assessment, and criteria for entrustment decisions (Figure 1)

| Training (sub)Program: Propofol-based deep sedation for a short (< 30 min), painful procedure in ASA 1 or 2 adult emergency department patients | |
| --- | --- |
| Clinical EPAs | DESCRIPTION |
| Perform a presedation assessment. | - Establish a trustful and professional relationship - Perform a relevant medical history, anesthesia/sedation history, airway evaluation, physical examination - Determine the sedation risks (ASA classification, airway characteristics, allergies) - Identify and address patient concerns and expectations - Inform the patient about sedation and procedure - Obtain informed consent |
| Prepare the procedure room and the team for deep sedation. | - Prepare medications - Create a calm environment; avoid unexpected events - Communicate the procedural plan with all involved and discuss roles - Make sure that all necessary materials and staff are readily available |
| Prepare the patient for deep sedation. | - Maintain a trustful and professional relationship - Establish vascular access - Ensure adequate analgesia - Start essential monitoring (oximetry, capnography, ECG, blood pressure) - Position the patient according to procedural needs |
| Initiate deep sedation. | - Administer the correct initial dose of propofol - Identify and manage changes in vital parameters (oximetry, capnography, ECG, blood pressure) - Maintain a calm and patient-centered environment - Identify when sedation is sufficient to start the procedure |
| Maintain an adequate level of sedation and analgesia. | - Adjust doses of propofol and analgesia in order to optimize patient comfort while assuring a patent airway and spontaneous ventilation. - Continue to monitor and manage changes in vital sign parameters |
| Organize the recovery following deep sedation. | - Continue to monitor and manage changes in vital sign parameters until patient is fully awake. - Manage postprocedural pain - Provide post discharge advice (pain management, follow-up, contact details for questions) |
