# Supplementary material for: Procedural sedation competencies: a review and multidisciplinary international consensus statement on knowledge, skills, training, and credentialing
Source: Br J Anaesth. 2024 Sep 25;134(3):817–29. doi: 10.1016/j.bja.2024.07.036 (PMC11867087; doi:10.1016/j.bja.2024.07.036)
Supplement: Multimedia component 2 [file mmc2.docx]

**Supplementary material 2: Tracking and Reporting Outcomes of Procedural Sedation Tool**
